# An unanswered question in pediatric urology: the post pubertal persistence of prepubertal congenital penile curvature correction by tunical plication

**DOI:** 10.1590/S1677-5538.IBJU.2017.0055

**Published:** 2017

**Authors:** Ünsal Ozkuvanci, Orhan Ziylan, M. Irfan Dönmez, Omer Baris Yucel, Tayfun Oktar, Haluk Ander, Ismet Nane

**Affiliations:** 1 Department of Urology, Istanbul Faculty of Medicine, Istanbul University, Istanbul, Turkey

**Keywords:** Penile Diseases, Penile Induration, Circumcision, Male

## Abstract

**Objective:**

The aim of this study is to analyze post pubertal results of pre pubertal tunica albuginea plication with non-absorbable sutures in the correction of CPC.

**Materials and Methods:**

The files of patients who underwent tunica albuginea plication without incision (dorsal/lateral) were retrospectively reviewed. Patients younger than 13 years of age at the time of operation and older than 14 years of age in November 2015 were included. Patients with a penile curvature of less than 30 degrees & more than 45 degrees and penile/urethral anomalies were excluded. All of the patients underwent surgery followed by circumcision.

**Results:**

The mean age of patients at the time of the operation was 9.7 years (range, 6-13 years). The mean degree of ventral penile curvature measured during the operation was 39 degrees while it was 41 degrees in the lateral curvatures. All of the patients were curvature-free at the end of the operation. At the time of the follow-up examination, the mean age was 16.7 years (range, 14-25 years). Six patients had a straight (0-10 degrees) penis during erection and seven patients had recurrent penile curvatures ranging from 30 to 50 degrees.

**Conclusion:**

Pre pubertal tunica albuginea plication of congenital penile curvature (30-45 degrees) with non-absorbable sutures performed without incision is a minimal invasive method especially when performed during circumcision. However, recurrence might be observed in half of the patients after puberty.

## INTRODUCTION

Congenital penile curvature (CPC) is a condition in which the erect penis is not straight and there are no urethral or penile anomalies such as epispadias or epispadias. Curvature is typically ventral, but it can also be dorsal, lateral or mixed (1). The prevalence of CPC ranges between 0.040.6% (2, 3). Skin tethering, abnormal Buck's or Dartos fascia, corporeal body disproportion, and rarely, short urethra are thought to play a role in the etiology (4). CPC may be recognized in early childhood by parents during morning erections, but it is more often diagnosed during puberty when the erections become frequent and the penis enlarges. CPC might decrease the individual's quality of life after adolescence by causing esthetic, functional, and psychological problems.

Currently, the EAU congenital penile curvature guidelines endorses post pubertal surgical correction as the mainstay treatment, while EAU pediatric urology guidelines indicates surgical correction of CPC over 30 degrees without identifying any age interval (5, 6). In a survey among American pediatric urologists, surgery has been shown to be recommended in patients with a degree of curvature greater than 20-30 degrees and in patients who feel the appearance of the penis is not esthetic during erection (7). However, the optimal technique and timing for surgery are controversial. In addition, there is limited data regarding the long-term follow-up of those patients. Our aim was to analyze the post pubertal persistence of CPC correction with tunica albuginea plication using non-absorbable sutures performed in children at prepubertal age.

## MATERIALS AND METHODS

After obtaining permission from the local board, the files of patients who underwent circumcision and congenital penile curvature correction between 1991 and 2012 were retrospectively reviewed. Patients who were found to have moderate (30-45 degrees) CPC during surgery were analyzed. Children aged younger than 13 years at the time of operation and older than 14 years in November 2015 were included. The exclusion criteria were having a penile curvature less than 30 degrees and more than 45 degrees after degloving, having undergone other correction techniques including plication with incision or Nesbit, and presence of penile or urethral anomalies (urethral hypoplasia and divergence of corpus spongiosum). Operation reports were reviewed in order to obtain per operative curvature specifications (laterality and degree).

**Figure 1:**
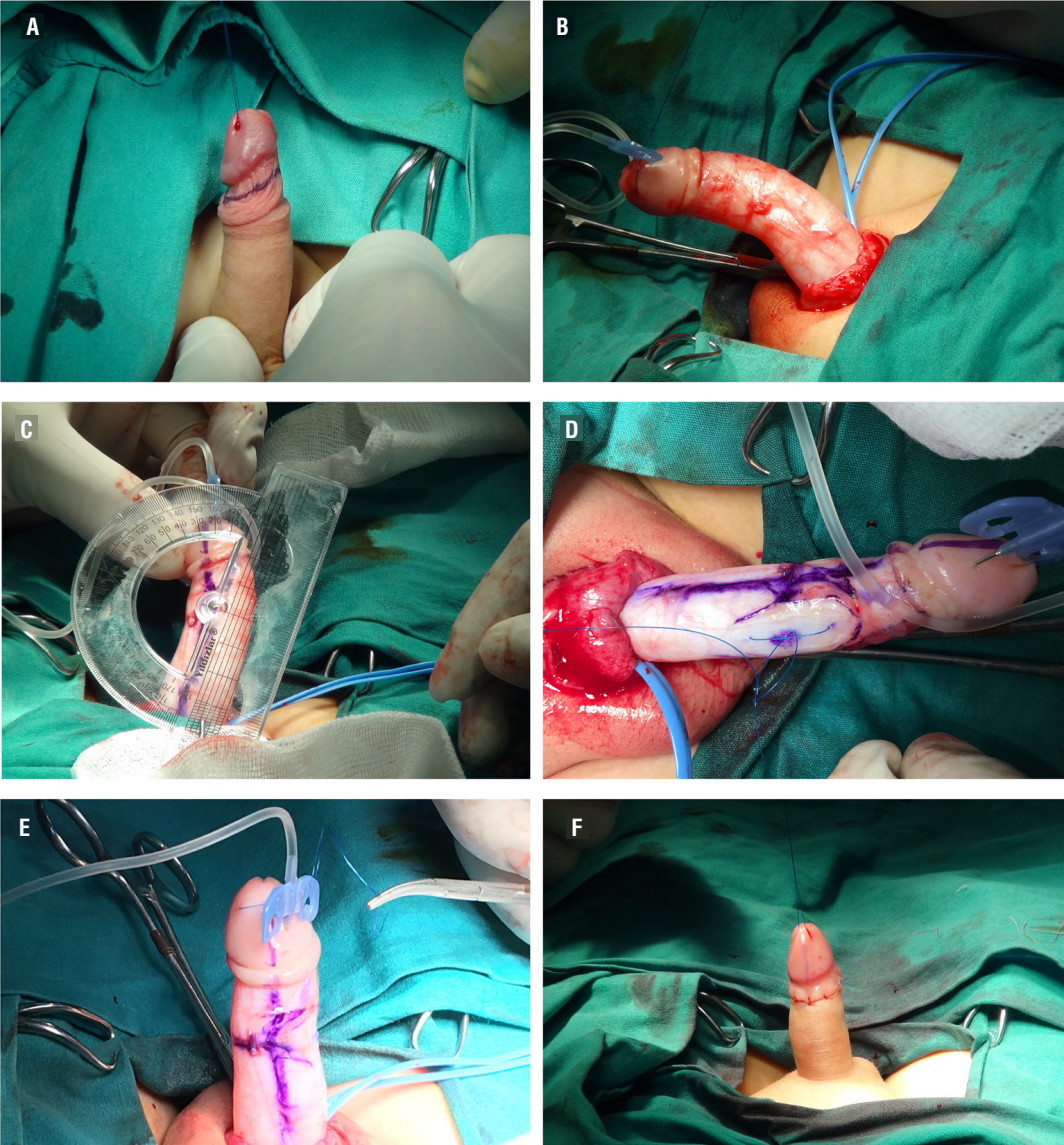
a) Marking prior to degloving of the penis; b) Artificial erection using saline; c) Measurement of the curvature using a goniometer; d) Correction of curvature by tunical plication; e) Control of curvature after plication; f) Circumcision.

All patients underwent tunica albuginea plication with non-absorbable sutures for patients with disproportional corpora cavernosa by one of two surgeons (OZ, TO), followed by circumcision. During the surgical procedure, a urethral catheter was inserted to assure the integrity of the urethra. Following a complete penile degloving and even release of the Dartos fascia until Buck's fascia, an artificial erection was obtained using saline injection after placing a proximal tourniquet. Afterwards, curvature degree was measured using a goniometer. The contralateral side to the maximal curvature of the corpus cavernosum was marked. Upon opening Buck's fascia, a 4.0/5.0 non-absorbable inverted suture was placed in the 12 o'clock position while preserving the dorsal vein in the ventral curvature or on the opposite side of the bend in the lateral curvature (Figure-1). Afterwards, straightening of the penis was checked again and residual curvature less than 10 degrees was accepted as successful. If the first plication was not sufficient to correct the curvature, additional plication sutures were placed where necessary. The procedure was finished after circumcision using the sleeve method. A self-adhesive dressing was applied for two days after the procedure. The urethral catheter was left in situ for one day.

Early postoperative results were obtained from patient files. Furthermore, patients were contacted by telephone and requested to attend a follow-up examination. The parents were consulted, and approval of the patients and their parents were both obtained. After physical examination, patients were questioned about what they thought of their current penile curvature. In addition to this, we also asked them to send us digital photos of the erect penis taken from the side and from the top. Photos were digitally reviewed and the degree of penile curvature was measured on the digital picture as the angle between the perpendicular lines originating from the glans and base of the penis. Preoperative and current curvature degrees of all patients were compared.

## RESULTS

A total of 13 patients who underwent prepubertal CPC correction and circumcision between 1991 and 2012 were studied. The mean age at the time of operation was 9.7 years (range, 6-13 years). All patients were admitted for circumcision and correction curvature at the same time. The mean ventral and lateral curvatures were 39 degrees and 41 degrees, respectively. Among these patients, nine had ventral curvatures, one had a ventral and a right lateral curvature, one had a left lateral curvature, and two had right lateral curvatures. Seven patients underwent one-suture midline dorsal plication, two had two-suture lateral plication, one had one-suture lateral plication, one had triple-suture lateral plication, one had two-suture midline dorsal plication, and another had a two-suture midline dorsal plication and a triple-suture lateral plication (Table-1). The mean operation time was 42 minutes (range, 29-51 minutes). All of the patients were curvature-free at the end of the operation. Furthermore, there were no signs of recurrence at the early postoperative control (3-6 months). The mean age at the time of examination was 16.7 years (range, 14-25 years) indicating a mean of 7 years of follow-up including the fastest growing interval of penis.

**Table 1:** Summary of the patients.

	Age at operation (years)	Per operative curvature	Operation type	Age at final follow-up (years)	Current curvature
**#1**	12	Ventral 45°	OMDP	25	Straight
**#2**	6	Ventral 30° + Right 45°	TMDP + ThLP	16	Straight
**#3**	11	Left 30°	OLP	16	Straight
**#4**	7	Ventral 30°	OMDP	17	Straight
**#5**	8	Ventral 45°	OMDP	17	Straight
**#6**	12	Ventral 45°	OMDP	15	Ventral 10°
**#7**	8	Ventral 45°	TMDP	14	Ventral 45°
**#8**	9	Ventral 45°	OMDP	15	Ventral 30°
**#9**	13	Ventral 45°	OMDP	17	Ventral 30°
**#10**	10	Right 45°	TLP	18	Right 50°
**#11**	7	Ventral 30°	OMDP	14	Ventral 30°
**#12**	11	Right 45°	TLP	17	Right 30°
**#13**	8	Ventral 30°	TMDP	16	Ventral 40°

**OMDP =** One-suture midline dorsal plication; **TMDP =** Two-suture midline dorsal plication; **TLP =** Two-suture lateral plication; **ThLP =** Triple-suture lateral plication; **OLP =** One-suture lateral plication

All follow-up photographs were digitally assessed in terms of curvature. Five patients had straight penises, one patient had less than 10 degrees of curvature, and the remaining seven patients had recurrent curvatures varying between 30-50 degrees. None of the patients had postoperative complications of wound healing, hemorrhage or urethral damage.

Patients were questioned about their penile perception. None of the patients reported erectile dysfunction. Furthermore, none of the patients reported feeling suture bumps although they were palpable in every patient during physical examination. Patients without recurrent curvature at the follow-up visit reported neither penile discomfort in erection nor penile deformity. However, every patient with recurrent curvature stated that they were unhappy with the appearance of their penis.

## DISCUSSION

The current literature does not provide comprehensive knowledge about the natural history of congenital penile curvature. As a result, there is no consensus/algorithm regarding technique and timing of the operation.

The process of penis development begins in the fetal period and continues until the end of puberty. Therefore, some authors suggest that penile correction surgeries should be performed after puberty (8), claiming that penile curvature can only be completely assessed after puberty (9).

On the other hand, it is known that having normal appearing external genitalia during puberty is necessary for healthy psychosexual development. The delay in the diagnosis of CPC has negative consequences on psychosocial development and quality of life (10). As a result, it is recommended to correct penile abnormalities at an early age (6-12 months) before sexual awareness begins (11). In our daily practice, patients/parents request surgery for CPC at school age, concomitantly with circumcision.

There are several methods for the correction of CPC, but none of these techniques is considered as a gold standard. Stepwise correction of anatomic abnormalities is recommended. The method of surgery is chosen according to the degree of curvature measured after the penis has been degloved. The two main methods are shortening and lengthening procedures. Lengthening is performed via urethral mobilization, corporotomy, and grafting of tunica albuginea of the curvature side, whereas shortening is achieved through various methods of plication and excision of the contralateral side. According to a survey of pediatric urologists, shortening procedures are recommended for mild or moderate curvatures, whereas lengthening procedures are recommended for severe curvatures (7).

Lengthening techniques are definitely more invasive than shortening techniques. Those who favor lengthening procedures consider that the pathology belongs to the shorter side, and therefore they find lengthening more logical (12). The authors proposed that most ventral curvatures develop due to urethral disorders. Chordee correction was possible by mobilization of urethra after penile degloving in 76% of cases, and dorsal plication after urethral mobilization in 8%. Only 16% required division/resection of hypoplastic urethra. None of them had residual chordee in a follow-up period of 6 months-3 years (mean: 26 months).

However, recent histopathologic studies have revealed that CPC is caused by abnormal development of the tunica albuginea of the corpora cavernosa and increased elasticity of tunica on the longer side, rather than urethral anomalies or short urethrae (13). In cases with ventral curvatures, asymmetric corporal development is responsible and the urethra is mostly normal, but in cases with lateral, dorsal or mixed curvatures, the abnormal development is always on the convex side of the tunica albuginea. We believe that CPC secondary to urethral abnormalities is less frequent and should be considered as hypospadias variants and therefore, should be managed accordingly.

Shortening techniques such as the Nesbit procedure or 16-dot plication have been shown as effective in adult CPC correction, but the use of these techniques in children is questionable. Baskin et al. showed penile neurovascular anatomy in hypospadiac and normal penises, and they also defined their technique of avoiding neurovascular damage (14). Their technique is effective, simple, and less invasive. As a result, penile deglovingand dorsal midline plication has gained popularity over time in chordee correction, hypospadias repair, and CPC correction among pediatric urologists. In a report regarding the long-term efficacy of dorsal plication with 83 patients, only 13 (16%) had CPC. The authors reported that dorsal plication of the tunica albuginea with sutures to 11-12-1 o'clock positions were successful in all cases and no recurrence was reported during a six-year follow-up period (15). However, it should be noted that the mean age at the time of surgery was 1.8 years and the mean follow-up period was 6 years, which does not include the fastest development period of the penis. Additional studies have reported good outcomes of dorsal plication without complications among the pediatric age group, whereas follow-up data after puberty is missing. Midline plication was performed for 43 children with penile curvatures. Only six had isolated penile curvatures and midline plication was successful for mild and moderate curvatures during the 16-month follow-up period (16). In another study, the authors performed midline plication for seven of ten penile curvatures caused by corporeal disproportion and reported no recurrent curvature during a 14.8-month follow-up period (17). In summary, the number of cases is small and the period of follow-up is short and information pertaining to puberty is lacking in the studies mentioned above. In the unique study regarding the post pubertal outcomes of prepubertal correction of CPC, Dipaola et al. showed that Nesbit dorsal plication had excellent results in cases of hypoplastic Dartos fascia. Also, they reported a 36% failure rate in more severe forms such as deficiency of corpus spongiosum, hypoplasia of the Buck's and Dartos fascia. Thus, the authors concluded persistence is related to the severity of the curvature rather than the age at surgery (18).

In our study, we aimed to evaluate the post pubertal results of our patients who underwent prepubertal dorsal and/or lateral tunica albuginea plication with non-absorbable sutures without incision, concomitantly performed with circumcision. Short-term follow-up examinations of these patients revealed no signs of recurrence. However, in the late follow-up after puberty, 7 out of 13 patients had recurrent curvature that required surgery. It is not certain whether this was a problem related to the technique or the nature of an ongoing developmental process. Although patients who required more sutures in order to obtain a straight penis during surgery seemed to be prone to recurrence, the numbers of patients are not enough to make a solid statement.

The post pubertal results of lengthening or tunical incision/excision techniques in children are scarce and as such, it is not clear whether these techniques have better outcomes. However, it is clear that tunica albuginea plication with non-absorbable sutures without incision is the least invasive technique compared with its counterparts. Moreover, prepubertal correction with this technique may also eliminate psychosexual disorders that may arise from CPC in half of the patients.

We believe that prepubertal correction of moderate CPC (30-45 degrees) using tunica albuginea plication with non-absorbable sutures, especially during circumcision, remains an alternative. Our current practice is to offer this technique to our patients along with circumcision because circumcision is a ritual in our country. If the patient has already undergone circumcision, the correction of curvature may be postponed until the post pubertal period. Finally, parents should be counseled in terms of a roughly 50% recurrence rate after puberty.

The limitations of our study are its retrospective nature, the limited number of patients, lack of a comparative arm, and deficiency of a validated questionnaire and comparatively subjective assessment of patients via digital photography. The use of questionnaires such as the International Index of Erectile Function or Self-Esteem and Relationship Questionnaire were not applicable in our population because the mean age at the time of first intercourse in boys is 17 years in our country (19). The strengths of the study are the post pubertal outcomes of a single operation technique from a single center.

## CONCLUSIONS

Prepubertal tunica albuginea plication with non-absorbable sutures without incision for corporal disproportion can be performed along with circumcision in moderate CPC. However, recurrence might be observed in half of the patients after puberty. It is not certain whether this is a problem related to the technique or the nature of an ongoing developmental process. Prospectively designed, comparative, and longitudinal studies are needed to form firm conclusions.
